# Supplementary material for: A CRISPR Interference Screen of Essential Genes Reveals that Proteasome Regulation Dictates Acetic Acid Tolerance in Saccharomyces cerevisiae
Source: mSystems. 2021 Jul 27;6(4):e00418-21. doi: 10.1128/mSystems.00418-21 (PMC8407339; doi:10.1128/mSystems.00418-21)
Supplement: TABLE S1 [file msystems.00418-21-st001.docx]

**Table S1**. qPCR_primer_sequences

| Primer | Sequence |
| --- | --- |
| RPN9_fw | GCAGATCCCTCTTTGCATCC |
| RPN9_rev | TTCGGACAATTGGAACCACA |
| RPT4_fw | CACCCAACAGTCACACGAAC |
| RPT4_rev | TTATGGGCCTCCTGTTCAGG |
| GLC7_fw | AGATATTCCCGACGTTGGCT |
| GLC7_rev | GCCCTGCAAATCAACTCCAT |
| YPI1_1 fw | TCCTCTTCTTCCGGATCCTC |
| YPI1_rev | GGCGCCTTTCCAATCTTCTT |
| ACT1_fw | CTACGTTTCCATCCAAGCCG |
| ACT1_rev | ACACCATCACCGGAATCCAA |
| IPP1_fw | ACTTCCCAGGTCTGTTGAGG |
| IPP1_rev | CCGGAGAAGGCAAATTGGTT |
